# A Hospital-Based Study on Attitude and Knowledge of Blood Donation and Practice of Blood Donation Among People in Kabul City, Afghanistan

**DOI:** 10.1155/anem/4779459

**Published:** 2025-09-22

**Authors:** Ahmad Khan, Melanie M. Tidman, Hasibullah Najib

**Affiliations:** ^1^A.T. Still Health Sciences University, Kirksville, Missouri, USA; ^2^Blossom Health Care Center, Kabul, Afghanistan

**Keywords:** attitude, blood donation, developing countries, knowledge, practice

## Abstract

This hospital-based study assessed attitudes, knowledge, and practices regarding blood donation in Kabul City, Afghanistan, focusing on whether demographic factors influence donor behavior. Through a hospital-based approach, using a survey questionnaire, the study examined donors' attitudes, knowledge of blood donation, and practice of blood donation. The study was conducted at Blossom Healthcare Center in Kabul City, Afghanistan, from August 1, 2024, to February 30, 2025. A total of 66 people, who were above the age of 18, participated in the study. Of the 66 participants, 66.7% (44) were male. There was no significant association between the level of education, age, and blood donation practice. Of the 66 participants, 42.4% (28) had a history of blood donation, and the percentage of blood donation was lower in women. Restrictions on women's independent mobility may negatively affect their blood donation practice. In conclusion, this hospital-based study explored Afghans' attitudes and knowledge toward blood donation in Kabul City, Afghanistan. We noticed that the blood donation practice did not positively correlate with the participants' positive attitude toward blood donation.

## 1. Introduction

Access to blood transfusions, essential for treating traumatic injuries and obstetric bleeding, varies significantly between high-income and lower- and middle-income countries (LMICs) [1–4]. While 117 million units of blood are collected annually, nearly 50% comes from high-income nations, leaving LMICs, such as those in South Asia and Sub-Saharan Africa, with severe shortages [5, 6]. The World Health Organization recommends that each country aim for a donation rate of 1% of its population, but LMICs average only five units per 1000 people, compared to 31.5 units in higher-income countries [7, 8]. Strategies to improve blood donations in LMICs can include public awareness campaigns, enhancing blood storage infrastructure, donor retention programs, and international collaboration among nations to share best practices to increase the percentage of blood donors [7]. Addressing blood donation-related challenges can significantly enhance the availability of blood products in LMICs such as Afghanistan.

Understanding donor eligibility criteria for blood donation is essential for healthcare practitioners and may therefore impact the education provided to potential donors, influencing their attitudes, knowledge, and practices. Typically, individuals must be at least 18 years old to donate, although some nations allow 16- and 17-year olds to donate with informed consent, despite a heightened risk of vasovagal reactions among younger donors [8–10]. The upper age limit for donation generally ranges from 60 to 70 years [11–13]. However, evidence is emerging that supports the safety of older donors, who meet the health criteria, leading some countries to remove this limit altogether [11–13]. Furthermore, potential donors must be symptom-free from acute infections and meet the minimum weight requirements of 45 kg or 50 kg for donations of 350 mL or 450 mL, respectively [14–19]. Understanding blood donation eligibility criteria is vital for promoting a positive attitude toward blood donation and promoting public awareness and practices in lower-income or underserved communities.

Standard health criteria for blood donation influence donor attitudes, knowledge, and practices, ensuring the safety of both donors and recipients. Essential indicators for eligibility include a systolic blood pressure of 120–129 mmHg, a diastolic pressure of 80–89 mmHg, a pulse rate of 60–100 beats per minute with a regular rhythm, and an average body temperature of 37°C (98.6°F) [20, 21]. According to the World Health Organization, hemoglobin thresholds are set at 12.0 g/dL for nonpregnant women and 13.0 g/dL for men, with prescreening recommendations of 12.5 g/dL and 13.5 g/dL, respectively [22–24].

Blood donation rates in LMICs such as Afghanistan are low due to multiple factors, including limited awareness, health-related infrastructure challenges, cultural misconceptions and beliefs, and accessibility issues [25, 26]. Besides addressing these issues to improve blood donation rates, it is essential to investigate specific factors that might influence blood donation behaviors among people in Afghanistan. Our hospital-based study in Kabul focuses on assessing the attitudes, knowledge, and practices of outpatient populations regarding blood donation, aiming to provide evidence that informs targeted interventions to improve blood donation rates in the community. Therefore, it is crucial to understand the qualitative themes related to blood donation barriers in the community to develop effective strategies to increase the willingness to donate blood among an informed donor base, thereby improving the blood supply in healthcare settings in Afghanistan.

## 2. Materials and Methods

### 2.1. Purpose

It is essential to have a consistent flow of blood donation practice in a health system to meet the needs of the patient population. This hospital-based, qualitative study aims to assess the general awareness and attitude toward blood donation and understand the practice of blood donation in Kabul, Afghanistan. To this date, the practice of blood donation in Afghanistan has not been assessed in the literature. This study was applied to understand if there are any barriers to blood donation related to people's attitudes and knowledge in Kabul City, Afghanistan. This study can assist public health authorities in tailoring their practices to increase the number of blood donors to fill in the gaps related to attitudes, knowledge, and practice of blood donation among a selected population receiving care at a local hospital to identify qualitative themes associated with barriers to blood donation among the participants in the study.

### 2.2. Ethical Consideration

Blossom Healthcare Center has determined the study as exempt from IRB oversight (Reference number: BHCC-189-2024). The researcher explained the purpose and nature of the study, focusing on the participants' attitudes, knowledge, and practices regarding blood donation. The information would only be used for educational and public awareness, and their confidentiality and privacy would be maintained. A copy of the questionnaire was provided to the participants, and their questions were answered after they gave their informed consent. Participants who met the inclusion criteria were included in the study. Participants were given a study ID number.

### 2.3. Study Dates and Location

An anonymous hospital-based survey study was conducted in Blossom Health Care Center in Kabul City, Afghanistan, from August 1, 2024, to February 30, 2025.

### 2.4. Sample/Population

Culturally, people in Afghanistan are less familiar with research than those in Western countries, which can limit the number of participants in a study. Some researchers highlighted that new information can be generated after interviewing at least 20 people [26]. We projected to have at least 20 participants and 66 participants from this convenience sample engaged in the study.

The data were collected from participants at Blossom Healthcare Center in Kabul City, Afghanistan, using nonprobability convenience sampling, which indicates that participants were selected based on their availability and interest in participating in the research. Stable patients (vital signs within the standard limit, no acute distress, conscious state, and their laboratory tests were within an acceptable range to their primary provider) and patients' family members coming to the hospital were verbally asked during their visit by their primary care doctor if they were interested in participating in the study. The researcher explained the purpose of the study, stating that the data would be used solely for educational purposes to facilitate further research in the future and potentially improve the policy and practice of blood donation among Afghans in Kabul City, Afghanistan. Every participant was informed that the interview would last approximately 10 min and asked if they were ready and felt they could answer the questions. The researcher asked the question in a private exam room with optimal temperature, natural light, and minimal background distraction. Each participant was informed that they were not required to answer questions they did not wish to. They could leave the interview at any time if they received an emergency call or they did not wish to continue.

The sample size for our outpatient hospital-based study was a convenience sample determined by considering blood donation attitudes, knowledge, and practices of patients attending the clinic visit at a hospital in Kabul City, aligning with the study's objectives. The primary objective of this study was to assess the attitudes, knowledge, and practices related to blood donation among a specific population in Kabul City, Afghanistan. The focus of the study was to obtain insights into participants' perspectives and behaviors.

A nonprobability sampling method, specifically convenience sampling, was used due to practical constraints such as time, human resources, the underdeveloped culture of research in the community surrounding Kabul City, Afghanistan, and the need for immediate participant access in an outpatient hospital setting. The sample type has limitations regarding the generalizability of the findings. However, it was a workable method for gathering sufficient information in a timely manner with limited resources. The sample size of 68 participants was selected based on the expected effect size. In studies involving attitudes and practices, effect sizes can vary; thus, we used guidelines from previous literature, which suggest that larger sample sizes yield more reliable data. The standard threshold of 0.80 (80%) power with a significance level (alpha) of 0.05 was selected to detect the actual effect if it truly existed. Effect sizes can be calculated or estimated based on pilot studies or literature reviews.

The sample size of 68 also reflects practical constraints of patients seeking medical care at larger hospitals in Afghanistan, and the sample in our study was collected with such constraints in mind such as participant availability, consent, and time required for data collection. After evaluating these considerations and the expected outcomes of our analyses, we determined a sample size that, although not derived from a traditional statistical power analysis, was sufficient to explore significant trends and relationships within our emerging data. Our findings contribute valuable insights, even within the limitations of nonprobability sampling.

### 2.5. Inclusion and Exclusion Criteria

Participants at least 18 years old, born and living in Afghanistan, and who signed the informed consent were included in the study. People who were not Native Afghans indicated that those who were not born and living in Afghanistan, younger than 18 years old, Afghan residents who did not understand Pashto or Dari, or who had hearing issues, and medically unstable patients were excluded.

### 2.6. Interview Procedure

The interview was conducted with participants who met the inclusion criteria and provided informed consent. The questionnaire was created using a theoretical triangulation health belief model, social cognitive theory, and theory of planned behavior, comprised of 17 closed-ended questions on sociodemographics, attitudes toward voluntary blood donation, and knowledge of voluntary blood donation practice (see Appendix A) [27–29]. For construct validity, the questionnaire was shared with a focus group of experts in medicine and research from Afghanistan. The Lawshe Content Validity Index was used to assess the content validity of the questionnaire to determine that the questions were relevant to knowledge and attitude about blood donation, and the content validity index was calculated at around 0.79 [30]. The questionnaire was pretested in person and online with Afghan residents with different sociodemographic backgrounds (participants were volunteers above the age of 18 years old with varying levels of education and work experience) to assess the time length of the full interview to mark period participant fatigue and to assess sociolinguistic competence, making sure that the questions and responses are acceptable according to the cultural norms and the responses and the words in the questions and answers made sense to the respondent. Sociologistic competence is essential in a qualitative study when the aim is to collect data on the participant's beliefs, emotions, attitudes, and experiences [31]. During the pretest and this anonymous interview period, besides participants' age, gender, level of education, and type of residence, which were analyzed and reported in the category to maintain participants' confidentiality and privacy, no other identifiable information was collected. Participants were assigned a study ID number to maintain the confidentiality of their responses, and their surveys were encoded with this unique identifier.

### 2.7. Operational Definitions

Attitude toward blood donation indicates positive motives for donating blood, knowledge of blood donation refers to the amount of information that determines when someone can donate blood, and the practices of blood donation refer to whether the participant has ever donated blood. Secondary and below education encompasses the 6th–9th grade of school. Commercial centers, universities, major hospitals, and government institutes characterize urban residential populated areas. Rural areas are characterized by places on the outskirts of metropolitan areas, including agricultural lands and people living in small villages.

### 2.8. Statistical Methods

We entered the data manually into an Excel spreadsheet and then used SPSS Version 28 to compile and analyze the data. The primary investigator performed the data analysis. Data from the Excel sheet was transferred to SPSS Software Version 28 (Armonk, New York, USA) for analysis. We used the Pearson chi-square test to assess whether there is a significant correlation between blood donation practice and participants age, level of education, gender, and type of residence. Pearson's chi-square test was used to assess the statistical significance, and the statistical test was a priori to highlight the gender dynamics surrounding blood donation in what we classified as a male-dominant culture.

## 3. Results

**Figure 1:**
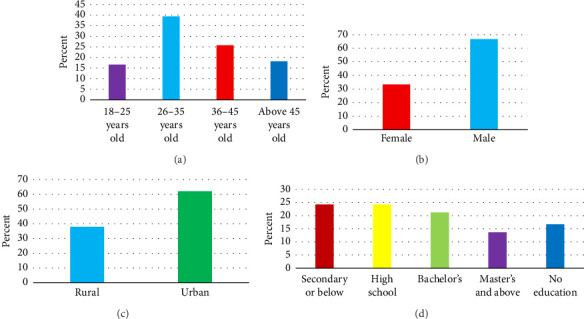
(a) Percentage of participants by age, (b) percentage of gender, (c) percentage of residence type, and (d) percentage of level of education.

We enrolled 66 participants between 18 and over 45 years old. Of the 66 participants, 44 identified as males, and 22 participants identified as females. Participants' residential types included rural and urban. The majority, 41 (62.1%), were residents of the urban areas. Their educational backgrounds included secondary school or below, high school, bachelor's, master's, and above. Notably, 16 (24.2%) of the participants were high school graduates (Figure 1); (a) percentage of participants by age, (b) percentage of gender, (c) percentage of residence type, and (d) percentage of level of education.

The chi-square test and associated measures were used to analyze the data to assess the associations between blood donation practices and participants' demographic variables, involving 66 valid cases.

### 3.1. Blood Donation Practices Association With Age

The chi-square test results for age indicated a Pearson chi-square value of 6.387 with 3 degrees of freedom and a *p* value = 0.094, indicating that there was no statistically significant association between blood donation practices and participants' age. The likelihood ratio test was consistent with this finding, with a value of 7.420 and a *p* value of 0.060, suggesting a weak association. In terms of effect size, Phi and Cramer's *V* are 0.311, indicating no significant association.

### 3.2. Blood Donation Practices Association With Gender

In contrast, the analysis of blood donation practices about gender showed a statistically significant association. The Pearson chi-square value is 7.940 with 1 degree of freedom, and a *p* value of 0.005. The likelihood ratio (value of 8.479, *p* value = 0.004), linear-by-linear association (value of 1.797, *p* value = 0.005), and the Phi coefficient of −0.347 and Cramer's *V* of 0.347 indicated that gender had a significant role in blood donation practices among participants.

### 3.3. Blood Donation Practices Association With Attained Education

The association between blood donation practices and attained education showed that the Pearson chi-square value is 8.581 with 4 degrees of freedom and a *p* value of 0.072, indicating a statistically nonsignificant association, and the linear-by-linear association was not significant, with a *p* value of 0.397.

### 3.4. Blood Donation Practices Association With Residence Type

The Pearson chi-square value was 11.504, with 1 degree of freedom, and a *p* value of 0.001, indicating a statistically significant association. The likelihood ratio aligned with this result, with a *p* value of 0.001. The linear-by-linear association indicated a *p* value of > 0.001, and the Phi coefficient of −0.417 and Cramer's *V* of 0.417 indicate a moderate to strong negative association, suggesting that residence type had a significant influence on blood donation practices (Table 1).

## 4. Discussion

Blood donation in LMICs is limited, and these countries do not meet the minimum threshold to meet their basic needs for blood products, resulting in a demand that cannot be met. Afghanistan is a lower-income country, and our sample revealed that the percentage of blood donors was lower in female genders and rural areas. Although we observed that 62 (93.9%) of the participants in the sample expressed a willingness to donate blood, in practice, 28 (42.4%) reported having actually donated blood. Our findings are consistent with study results from Bangladesh and India [32]. The number of voluntary blood donors is lower in Bangladesh, 4 per 1000 people, and in India, it is 5 per 1000 people, whereas, in higher-income countries, the number is higher. In Japan, the number of voluntary blood donors is 70 per 1000; in Switzerland, the number is 113 per 1000 people [32].

Factors leading to lower voluntary blood donation levels can vary from country to country. Still, in many lower-income countries, besides the socioeconomic challenges, people have limited knowledge of the safety of donating blood [32]. In a study conducted at a university in Bangladesh, 82% of students exhibited a positive attitude toward blood donation, which is consistent with the findings of our study that 62 (93.9%) of the participants reported a positive attitude toward blood donation. However, only 16% of the students in Bangladesh had a history of blood donation [33], and in our sample, the percentage of blood donors was 28 (42.4%). We found no significant correlation between blood donation practice and the participants' educational level. A cross-sectional study conducted at Benin Teaching Hospital supported our findings by also indicating that there was no significant correlation between the level of education and blood donation practice [34].

Research in multiple countries regarding blood donation indicates that the participation of women is less than that of men. Study results indicated donations for women in Ethiopia are at 12.1%, Netherlands at 18.7%, China at 18.6%, Iraq at 0.3%, Qatar at 2.6%, Turkey at 11%, and Cameroon at 19.5% [35–41]. Our findings are consistent with these study results, as we found that the percentage of female blood donors was lower than that of males with a significant correlation between the practice of blood donation and male gender. A study's results in Iran highlighted that women had adequate awareness of the importance of blood donation [42]. Some reasons for lower female participation in the Iran study were anemia, lack of time, fear, difficulties accessing donation centers, and lack of family permission [42]. In Afghanistan, due to sociocultural restrictions, women do not have the privilege and freedom of movement and access to health practices that men do, which can negatively affect their participation and awareness of the practice of blood donation. Further research in this area may be helpful to investigate whether social restrictions on women have negatively impacted blood donation practices in Afghanistan.

Studies that investigate how people perceive blood donation in lower-income countries and what the precursors are to their perceptions and willingness to donate blood are limited [43]. In a large-scale study in the United States of America, participants indicated that their motivation for blood donation was that donating blood is beneficial for the donor's health [44]. Primary motives reported among participants at King Abdul-Aziz University, Rabigh campus, Jeddah, indicated that participants felt they were assisting families (30%), saving other lives (28%), religious reasons (20%), and altruism (12%) [45]. In our sample, 60 (90.1%) indicated that blood donation is beneficial to the health of the donor and the recipient.

### 4.1. Limitations

Conducting research on attitudes, knowledge, and blood donation practices in Kabul City, Afghanistan, with a limited number of female participants, limits the generalizability of this study. Women's attitudes, knowledge, and practices of blood donation can be influenced by cultural and socioeconomic factors distinct from those affecting men. The limited number of women in our study may have resulted in the missing of critical insights regarding their attitudes, knowledge, and practice of blood donation.

Also, a dominant male perspective, which is experienced in Afghanistan, may lead to biased findings that reflect the views and experiences of men rather than a balanced understanding of attitudes and knowledge about blood donation between males and females in Kabul City, Afghanistan. This gender bias could lead to the stereotyping of women's roles on the part of providers in healthcare settings.

To address the limitations of the lower number of women in healthcare practices and other research, it is essential to conduct studies to highlight the potential barriers to elucidate why women are not donating blood and then prioritize strategies that enhance the participation of women in donating blood and blood products in their local communities. Campaigns to increase awareness of blood donation for families and giving women more freedom to share their insights or ask questions about blood donation without fear of stigmatization may prove beneficial.

## 5. Conclusions

In conclusion, this hospital-based study explored Afghans' attitudes and knowledge toward blood donation in Kabul City, Afghanistan. We identified that the actual practice of blood donation was not reflected in the participants' positive attitudes toward blood donation. Also, the practice of blood donation was lower in rural areas and among women. We recommend further research to assess the factors that contribute to the lower rate of blood donation, even in the presence of a positive attitude toward blood donation and with an apparent amount of adequate knowledge of blood donation. Further research and large-scale studies are critical to addressing barriers related to blood donation based on local evidence, which will help tailor awareness campaigns and community engagement strategies to overcome these barriers in Afghan communities, enhance the positive perception of blood donation, and increase the rates of blood donations to meet an ever-expanding need for blood and blood products in healthcare settings in lower-income countries.

## Figures and Tables

**Table 1:** Associations between blood donation practices and demographic variables among participants.

		Value	Degree of freedom	Approximate significance
Blood donation practices association with age				
Pearson chi-square		6.387^a^	3	0.094
Likelihood ratio		7.420	3	0.060
Linear-by-linear association		1.797	1	0.180
Nominal-by-nominal symmetric measure	Phi	0.311		0.094
	Cramer's *V*	0.311		0.094

Blood donation practices association with gender				
Pearson chi-square		7.940^b^	1	0.005
Likelihood ratio		8.479	1	0.004
Linear-by-linear association		1.797	1	0.005
Nominal-by-nominal symmetric measure	Phi		−0.347	0.005
	Cramer's *V*		0.347	0.005

Blood donation practices association with attained education				
Pearson chi-square		8.581^c^	4	0.072
Likelihood ratio		9.594	4	0.048
Linear-by-linear association		0.719	1	0.397
Nominal-by-nominal symmetric measure	Phi	0.361		0.072
	Cramer's *V*	0.631		0.072

Blood donation practices association with residence type				
Pearson chi-square		11.504^d^	1	< 0.001
Likelihood ratio		12.354	1	< 0.001
Linear-by-linear association			1	< 0.001
Nominal-by-nominal symmetric measure	Phi	−0.417		< 0.001
	Cramer's *V*	0.417		< 0.001
Number of valid cases		66		

^a^1 cell (12.5%) has an expected count of less than 5. The minimum expected count is 4.67.

^b^0 cells (0.0%) have an expected count of less than 5. The minimum expected count is 9.33.

^c^2 cells (20.0%) have an expected count of less than 5.

^d^0 cells (0.0%) have an expected count of less than 5. The minimum expected count is 10.61.

**Table 2:** Summary of survey responses on blood donation knowledge, attitude, and practice.

Question	Response	Count	Percentage
A person can donate around 450 cc of blood at a time	Yes	45	68.2
	No	16	24.2
	Not sure	5	7.6

A healthy person can donate blood 6 times in a year	Yes	32	48.5
	No	29	43.9
	Not sure	5	7.6

Do you know the age limit, body weight, and hemoglobin level to donate blood?	Yes	45	68.2
	No	15	22.7
	Not sure	6	9.1

People who have health issues (e.g., hepatitis B and hepatitis C) can donate blood	Yes	25	37.9
	No	38	57.6
	Not sure	3	4.5

Can a person get an infection by receiving a blood transfusion?	Yes	47	71.2
	No	16	24.2
	Not sure	3	4.5

Does blood donation have some health benefits?	Yes	60	90.9
	No	4	6.1
	Not sure	2	3

Is free blood donation better than donating blood for money?	Yes	59	89.4
	No	6	9.1
	Not sure	1	1.5

Does blood donation have a bad effect on health? Are you afraid of blood donation?	Yes	40	60.6
	No	22	33.3
	Not sure	4	6.1

Are you willing to donate blood?	Yes	62	93.9
	No	2	3
	Not sure	2	3

Should only adult males donate blood?	Yes	41	62.1
	No	22	33.3
	Not sure	3	4.5

Should blood only be donated to family members and friends?	Yes	35	53
	No	29	43.9
	Not sure	2	3

Do you know your blood group?	Yes	57	86.4
	No	8	12.1
	Not sure	1	1.5

Have you ever donated blood?	Yes	28	42.4
	No	38	57.6
	Not sure	0	0

## Data Availability

The data of this study are available upon request. Please contact the corresponding author at sa205310@atsu.edu for access to the dataset used in this study. Data will be provided in accordance with ethical guidelines.

## Appendix A

Survey questionnaire related to blood donation knowledge, attitude, and practice (Table A1).

The questionnaire was created by an Afghan focus group using a theoretical triangulation health belief model, social cognitive theory, and theory of planned behavior, comprised of 17 closed-ended questions on sociodemographics, attitudes toward voluntary blood donation, and knowledge of voluntary blood donation practice.
